# Gestational weight gain and group prenatal care: a systematic review and meta-analysis

**DOI:** 10.1186/s12884-018-2148-8

**Published:** 2019-01-09

**Authors:** Michelle A. Kominiarek, Adam K. Lewkowitz, Ebony Carter, Susan A. Fowler, Melissa Simon

**Affiliations:** 1 Department of Obstetrics and Gynecology, Division of Maternal-Fetal Medicine, Northwestern University Feinberg School of Medicine, 250 East Superior Street, Suite 05-2175, Chicago, IL 60611 USA; 2 Department of Obstetrics and Gynecology, Division of Maternal-Fetal Medicine, Washington University School of Medicine, St. Louis, MO USA; 3 Brown School Library, Washington University in St. Louis, St. Louis, MO USA; 4 Department of Obstetrics and Gynecology, Northwestern University Feinberg School of Medicine, Chicago, IL USA

**Keywords:** Group prenatal care, Gestational weight gain, Perinatal outcomes

## Abstract

**Background:**

Group visits for chronic medical conditions in non-pregnant populations have demonstrated successful outcomes including greater weight loss compared to individual visits for weight management. It is plausible that group prenatal care can similarly assist women in meeting gestational weight gain goals. The purpose of this study was to evaluate the effect of group vs. traditional prenatal care on gestational weight gain.

**Methods:**

A keyword search of Medline, Embase, Scopus, Cochrane Database of Systematic Reviews, Database of Abstracts of Reviews of Effects, Cochrane Central Register of Controlled Trials, clinicaltrials.gov, and Google Scholar was performed up to April 2017. Studies were included if they compared gestational weight gain in a group prenatal care setting to traditional prenatal care in either randomized controlled trials, cohort, or case-control studies. The primary and secondary outcomes were excessive and adequate gestational weight gain according to the Institute of Medicine guidelines. Heterogeneity was assessed with the Q test and I^2^ statistic. Pooled relative risks (RRs) and confidence intervals (CI) were reported with random-effects models from the randomized controlled trials (RCT) and cohort studies.

**Results:**

One RCT, one secondary analysis of an RCT, one study with “random assignment”, and twelve cohort studies met the inclusion criteria for a total of 13,779 subjects. Thirteen studies used the CenteringPregnancy model, defined by 10 sessions that emphasize goal setting and self-monitoring. Studies targeted specific populations such as adolescents, African-Americans, Hispanics, active-duty military or their spouses, and women with obesity or gestational diabetes. There were no significant differences in excessive [7 studies: pooled rates 47% (1806/3582) vs. 43% (3839/8521), RR 1.09, 95% CI 0.97–1.23] or adequate gestational weight gain [6 studies: pooled rates 31% (798/2875) vs. 30% (1410/5187), RR 0.92, 95% CI 0.79–1.08] in group and traditional prenatal care among the nine studies that reported categorical gestational weight gain outcomes in the meta-analysis.

**Conclusions:**

Group prenatal care was not associated with excessive or adequate gestational weight gain in the meta-analysis. Since outcomes were overall inconsistent, we propose that prenatal care models (e.g., group vs. traditional) should be evaluated in a more rigorous fashion with respect to gestational weight gain.

**Electronic supplementary material:**

The online version of this article (10.1186/s12884-018-2148-8) contains supplementary material, which is available to authorized users.

## Introduction

Since pregnancy is a time when women may be motivated to improve their health behaviors, it is often considered the optimal time to intervene on health behaviors such as eating habits and physical activity so that gestational weight gain goals are met and perinatal outcomes optimized [1]. Excessive gestational weight gain is positively correlated with postpartum weight retention and is a predictor of long-term, higher body mass index in women and their offspring [2–4]. Proposed long-term metabolic consequences of excessive gestational weight gain for women include type 2 diabetes, cardiovascular disease, and metabolic syndrome [5]. Trends in increasing adult weights and excessive gestational weight gain in the United States over the past two decades have shifted the focus of gestational weight gain counseling so as to avoid excessive gestational weight gain. Nonetheless, according to a national study, nearly 50% of all women exceeded these goals in 2010–2011 [6]. As such, meeting gestational weight gain goals is important for women and their offsprings’ long-term health.

Although diet and exercise interventions can reduce excessive gestational weight gain by 20%, some critiques of these trials are that they failed to address the relationship between psychosocial factors (e.g., depression, body image, and social support) and gestational weight gain [7, 8]. Furthermore, the majority of the interventions were performed in individual sessions with 1:1 healthcare professional-participant settings. Group visits for weight management in non-pregnant populations have demonstrated successful outcomes including greater weight loss compared to usual care [9, 10]. It is plausible that group prenatal care can similarly assist women in meeting gestational weight gain goals. Evidence suggests that compared to those receiving traditional individual prenatal care, women who receive group prenatal care have lower rates of preterm birth and cesarean delivery and higher rates of breastfeeding and knowledge and satisfaction with prenatal care. However, these findings have not been consistent and the mechanism for the possible improvement in outcomes is unknown [11–14]. Sheeder et al. conducted a review of group prenatal care literature and cited only two studies about gestational weight gain with one reporting increased mean weight gain in group participants without regard to the pre-pregnancy body mass index and the other reporting no differences in responses to a question about whether women made health behavior changes “to gain an appropriate amount of weight” at the end of the study between group and non-group participants [15].

## Objective

Given the importance of meeting gestational weight gain goals for a woman’s long term health, the limitations of health behavior interventions to promote meeting gestational weight gain goals, and the limited evidence for group prenatal care and gestational weight gain outcomes, the objective of this study was to systematically review the literature to compare gestational weight gain in group and traditional prenatal care and evaluate group prenatal care for having excessive or adequate gestational weight gain with a meta-analysis.

## Methods

### Eligibility criteria, information sources, search strategy

The published literature was searched using strategies created by a medical librarian (S.A.F.) for the concepts of group prenatal care and gestational weight gain.(Additional file 1) These strategies were established using a combination of standardized terms and key words, and were implemented in Medline 1946-, Embase 1947-, Scopus 1823-, Cochrane Database of Systematic Reviews, Database of Abstracts of Reviews of Effects, Cochrane Central Register of Controlled Trials, clinicaltrials.gov 2000-, and Google Scholar. All searches were completed in April 2017. No database limits such as language or years were applied. All literature database and Google Scholar results were exported to EndNote.

### Study selection

Studies were included if they described a group prenatal care setting (exposure) and reported gestational weight gain (outcome), either as means or by adequacy of gestational weight gain categories, per the Institute of Medicine guidelines. We included original research studies such as randomized controlled trials or observational studies (retrospective cohort, case-control) with a comparison group of traditional or individual prenatal care. We excluded case reports, case series, review articles, studies without comparison groups, and studies published in languages other than English.

### Data extraction

The first and second authors (M.A.K., A.K.L.) screened the titles and abstracts and then retrieved the full-text articles if they appeared relevant or if there was uncertainty regarding the screening criteria. Full-text articles and abstracts (when full-text was not available) were independently reviewed for inclusion and exclusion criteria by the same authors (M.A.K., A.K.L.), who have expertise in prenatal care. Consensus was achieved between these two authors for included articles. For each article that met the inclusion criteria, study characteristics, participant demographics (age, parity, race-ethnicity, body mass index), study-specific inclusion criteria, setting, gestational weight gain definition, and outcome data relevant to gestational weight gain were abstracted and summarized. Specifications for body mass index (pre-pregnancy vs. first prenatal care visit measurement vs. not recorded) and gestational weight gain (difference between the pre-pregnancy vs. first prenatal visit and final prenatal visit vs. delivery weight vs. not recorded) were also abstracted. Categorization of gestational weight gain was defined by the 2009 Institute of Medicine guidelines per pre-pregnancy body mass index category (28–40 pounds for < 18.5 kg/m^2^, 25–35 pounds for 18.5–24.9 kg/m^2^, 15–25 pounds for 25.0–29.9 kg/m^2^, and 11–20 pounds for ≥30 kg/m^2^), if applicable, with the terms inadequate, adequate, or excessive [16]. We also noted whether or not gestational age at delivery was accounted for in the total gestational weight gain measurement and whether or not the total number of group or traditional prenatal care visits attended was reported.

### Study quality assessment

The quality of each study was assessed with the Down’s checklist which contains 27 questions pertaining to threats to validity including reporting, external validity, internal validity, confounding or selection bias, and power [17]. The Down’s checklist is validated for both randomized and observational studies. The maximum possible score was 28, indicating the highest quality study. We considered studies receiving the majority of the points available in at least four of the five categories of threats as high quality with scoring similar to O’Connor et al. (24–28 excellent, 19–23 good, 14–18 fair, and < 14 poor) [18]. Both M.A.K. and A.K.L. completed the quality rating form for each article, with E.C. resolving any discrepancies in scoring to achieve a final consensus score.

### Data synthesis

The primary outcome was the occurrence of excessive gestational weight gain as determined by body mass index and the Institute of Medicine guidelines, but we also evaluated the occurrence of adequate gestational weight gain. Data were analyzed with STATA (version 14, College Station, TX) using the METAN software package. Study heterogeneity was assessed with Cochran’s Q and Higgins I^2^ tests. Conservative significance thresholds of *P* < 0.1 for the Q tests or I^2^ > 30%, were used to test heterogeneity [19]. Relative risks (RR) were calculated with raw data from each study with a 95% confidence interval (CI) using raw data from each study. The DerSimonian-Laird random-effects models were used to pool data, regardless of whether there was evidence of statistical heterogeneity. RR for each categorical outcome were plotted graphically as forest plots. A sensitivity analysis by study quality with those studies at the top tertile of Down’s scores was also performed to assess the effect of these factors on our estimates. Publication bias was assessed graphically using funnel plots.

Institutional review board approval was not necessary for this study of de-identified data available in the public domain through prior publications. This systematic review was registered in PROSPERO on February 1, 2017 (#CRD42017056296). The Preferred Reporting Items for Systematic Reviews and Meta-Analyses (PRISMA) guidelines were followed for all aspects of reporting [20].

## Results

### Study selection

**Fig. 1:**
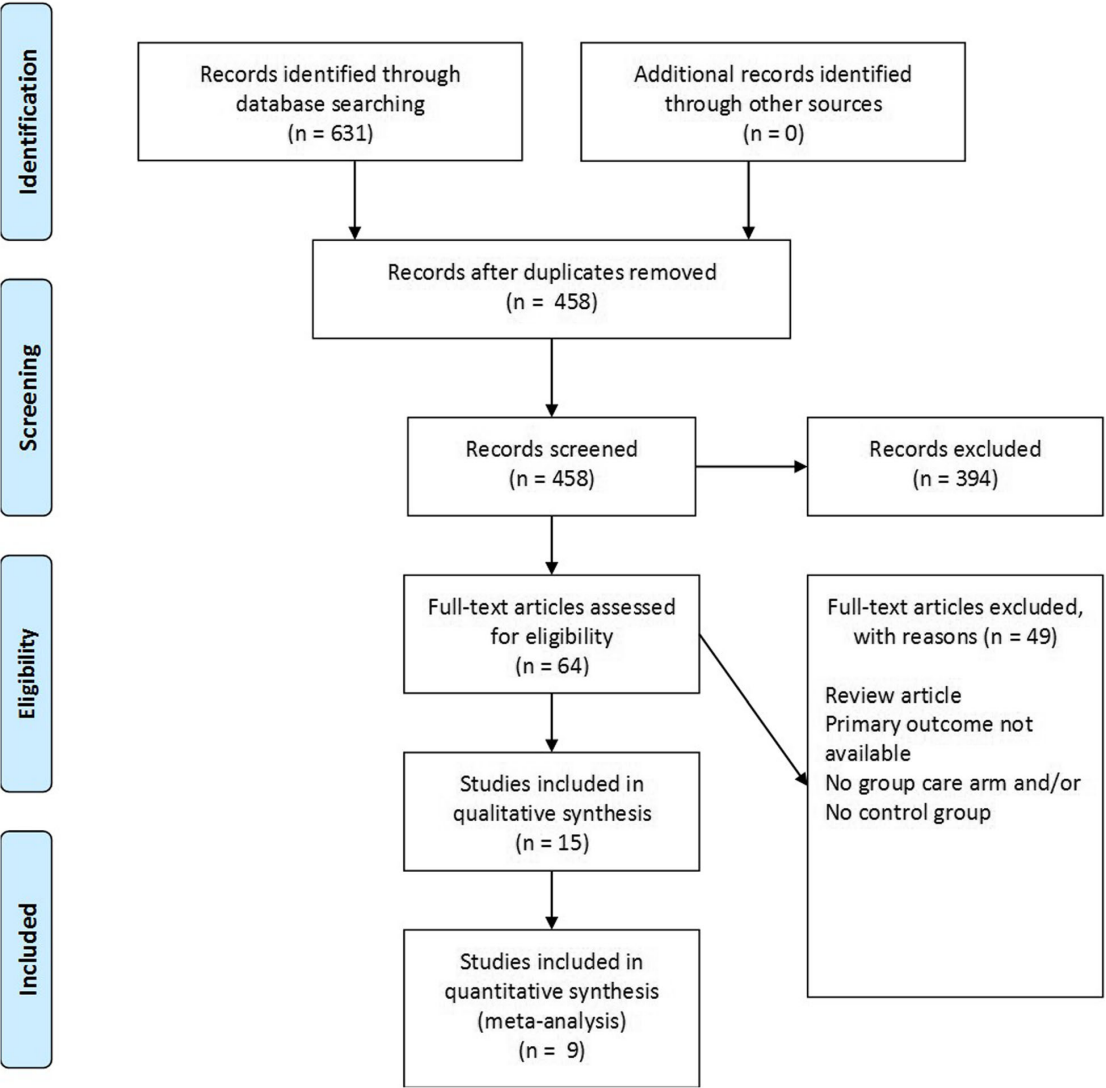
PRISMA Flow diagram for study selection

The initial electronic search found 631 results. One hundred twenty six duplicates were accurately identified and removed with the automatic duplicate finder in EndNote. An additional 47 duplicate citations were identified by hand and removed for a total of 458 unique citations.(Fig. 1) Titles and abstracts for each citation were reviewed and 64 full-text articles (or abstracts if full-text was not available) were screened according to the inclusion and exclusion criteria. Reference lists of excluded articles were reviewed to identify other relevant articles and no additional studies were found. A total of 49 studies were removed for no reported gestational weight gain outcomes, no group prenatal care arm, no control group, and/or review article only.

### Study characteristics

Fifteen studies met inclusion criteria, including one randomized controlled trial (RCT), one secondary analysis of an RCT, one study with “random assignment” and 12 cohort studies for a total of 13,779 subjects, 4243 (31%) in group and 9536 (69%) in traditional prenatal care. Table 1 describes the characteristics of studies meeting inclusion criteria. CenteringPregnancy™ group prenatal care was the most common prenatal care model used in included studies (*n* = 13). In CenteringPregnancy™, women receive prenatal visits, build relationships with other women, and gain knowledge and skills in pregnancy and childbirth in 10 sessions [21]. During the first group session, nutrition, including caloric requirements and macronutrient recommendations, is typically discussed. Women also receive a notebook that includes a food diary and a body mass index table labeled with the categories of normal, overweight, obese, and extreme obesity. Additionally, women chart their own weight over time in the notebooks. The curriculum overall encourages goal setting, including diet, exercise, and weight gain. None of the studies using the CenteringPregnancy™ model in this systematic review reported whether or not the content was specifically adapted to target weight gain. The two non-CenteringPregnancy™ models are described as follows. Harden et al. performed a mixed methods study on group-based lifestyle sessions within prenatal care with six-1 h group visits consisting of safe group exercises, nutrition education and demonstrations, and group activities such as goal setting [22]. Mazzoni et al. described a group prenatal care intervention consisting of four sessions for women with gestational diabetes [23]. Several of the other included studies describe an intervention that was targeted at specific populations such as adolescents [24, 25], African-Americans [26], Hispanics [27], active-duty military or their spouses [28, 29], women with obesity [22] and women with gestational diabetes [23, 30].

**Table 1:** Characteristic of studies comparing gestational weight gain in group and traditional prenatal care settings

Author Publication Year	Study Design Study Years	Country/Setting	Inclusion/Exclusion for Group Care at the site	Inclusion/Exclusion for the study of group care at the site	Participant demographics	BMI, GWG, and GWG goal definitions	Prenatal care model
Randomized Controlled Trials							
Kennedy 2011 [29]	RCT 2005–2007	US Military Naval Hospital in Pacific Northwest	< 16 weeks, ≥18 years, no severe medical problems, understand English	< 16 weeks, ≥18 years, no severe medical problems, understand English	58–60% white Mean age 25 years 45–59% nulliparas Mean BMI range 26.0–26.4 kg/m^2^at study baseline	BMI: not stated GWG: difference between time 1 (12–16 weeks) and delivery GWG goals: not stated	CP
Harden 2014 [22]	Feasibility pilot study with “random assignment” Years not stated	Country not stated Low-income families	Pre-pregnancy BMI ≥ 30 kg/m^2^, attended clinic that served low-income families, physician clearance, 21–35 years, < 21 weeks, no medical contraindication to physical activity, English speaking	Pre-pregnancy BMI ≥ 30 kg/m^2^, attended clinic that served low-income families, physician clearance, 21–35 years, < 21 weeks, no medical contraindication to physical activity, English speaking	61.5% Caucasian Mean age 22 years 100% obese with mean BMI 37.64 kg/m^2^	BMI: pre-pregnancy weight GWG: week 4,8,12,16,20 of intervention minus beginning of intervention GWG goals: 2009 IOM	Prenatal group visits in group-based lifestyle sessions
Magriples 2015 [25]	Secondary analysis from RCT 2008–2012	US 14 Community Health Centers and hospitals in New York City, New York serving low-income minorities	14–21 years, < 24 weeks, no severe medical problems, English or Spanish speaking	Excluded: multiple births, history of heart disease, CHTN, DM, no BMI data, missing or invalid weight data	63.5% Latina Mean age 19 years 85% nulliparas 12% underweight 53% normal 20% overweight 16% obese	BMI: self-reported pre-pregnancy weight GWG: not stated GWG goals: 2009 IOM	CP Plus (Structured reproductive health promotion activities during 4 of the 10 sessions including activities to improve sexual self-efficacy, HIV knowledge, interpersonal sexual communication, perceived risk, and social norms)
Cohort studies							
Klima 2009 [26]	Retrospective cohort 2004–2006	US Low-income Chicago, Illinois public health clinic, all women eligible for Medicaid	Continuing care at site, < 18 weeks, no high risk obstetric conditions	Exclusions: fetal demise < 28 weeks, delivered before attending 4 group or individual visits, delivery at other hospital	100% African-American Mean age range 20.8–22.1 years	BMI: not stated GWG: not stated GWG goals: not stated	CP
Holbrook 2010 [31]	Pilot chart review 2009	US Northern California	Not stated	Spanish-speaking	100% Hispanic	BMI: not stated GWG: first recorded weight to last recorded weight GWG goals: excessive defined as > 1 pound/week in the 3rd trimester	CP
Trudnak 2013 [27]	Retrospective cohort 2006–2010	US Southern Florida public health clinic	Spanish-speaking, Hispanic ethnicity	Initial visit completed before 4 months gestation, completed at least 50% of visits	> 90% Hispanic Mean age range 25–26.0 years 33–54% nulliparas 2% underweight 50% normal weight 33% overweight 13% obese 1% morbid obese	BMI: pre-pregnancy weight GWG: weight at last prenatal visit minus pre-pregnancy weight GWG goals: 2009 IOM	CP
Tanner-Smith 2014 [35]	Retrospective cohort 2008–2011	US Faith-based community health center in Southern metropolitan area	English speaking, no high risk conditions (prior CD, prior LBW, DM, SLE, heart disease, clotting problems, seizures, kidney disorder, cervical incompetence, mental health disorder)	Not stated	76% non-Hispanic black, 13% Latina, 11% White 88% public insurance Mean age 22 years Median gravidity 2 43% healthy weight 30% overweight 27% obese	BMI: pre-pregnancy weight GWG: last prenatal visit minus 1st prenatal visit GWG goals: IOM 2009 based on pre-pregnancy weight	CP
Zielinski 2014 [34]	Case-control retrospective 2010–2012	US CNM hospital-based practice in southwest Michigan	< 20 weeks, eligible for CNM care	< 20 weeks, eligible for CNM care	Mean age range 24–25 years 49% private insurance 78% white 4% underweight 41–42% normal 29–30% overweight 25% obese	BMI: self-reported pre-pregnancy weight GWG: not stated GWG goals: 2009 IOM	CP
Walton 2015 [28]	Retrospective cohort 2009–2013	US Military Naval Medical Center in San Diego, California in a tertiary care teaching hospital with outpatient clinics	Not stated	Included: Delivery at Naval Medical Center in San Diego, > 20 weeks at delivery Excluded: “late to care”, transfer of care, dependent daughters of active duty members	48% Caucasian Mean age range 25–26 years 57–75% nulliparas Mean range of pre-pregnancy BMI 24.3–25.0 kg/m^2^	BMI: pre-pregnancy weight GWG: not stated GWG goals: 2009 IOM	CP
Trotman 2015 [24]	Retrospective cohort 2008–2012	US MedStar Health Research Institute Washington, D.C. in a teaching urban hospital with adolescents	Not stated	11–21 years, No significant medical history or pregnancy complication requiring high risk care	96% African-American Mean age 17 years 70% nulliparas	BMI: not stated GWG: not stated GWG goals: 2009 IOM	CP
Mazzoni 2015 [23]	Prospective observational 2013–2014	US Hospital based clinic in Denver, Colorado affiliated with integrated safety net healthcare system	GDM, English or Spanish speaking, singleton	≥ 2 of the 4 group visits	Mean age 31 years 89% Hispanic Mean pre-pregnancy BMI range 30–33 kg/m^2^ 5–6% CHTN	BMI: pre-pregnancy weight GWG: not stated GWG goals: not stated	Group care for GDM at 26–32 weeks with 4 sessions
Brumley 2016 [36]	Retrospective cohort 2011–2013	US University of South Florida, academic center	Excluded: pregestational DM, CHTN, uncontrolled psychiatric disorders, multiple gestations, other major medical problems, > 20 weeks	≥ 3 group visits	62–76% white 57–79% nulliparas 2.1% underweight 60% healthy weight 22.6% overweight 15.4% obese	BMI: initial weight or self-reported pre-pregnancy weight if 1st visit occurred after the 1st trimester GWG: weight at last visit minus weight at 1st visit GWG goals: IOM, year not stated	CP
O’Donnell 2016 [32]	Retrospective cohort 2011–2014, abstract only	US University of California-San Francisco Medical Center, CA	Not stated	18–45 years, singleton, no fetal anomalies, no chronic medical problems, 1st prenatal visit < 20 weeks, attended ≥5 prenatal visits	Mean age 33 years 51% white, 25% Asian Mean pre-pregnancy BMI 24 kg/m^2^	BMI: self-reported pre-pregnancy weight GWG: weight at delivery minus self-reported pre-pregnancy weight GWG goals: 2009 IOM	CP
Schellinger 2016 [30]	Retrospective cohort 2010–2015	US Inner city county hospital in Indiana	GDM, English or Spanish speaking	≥ 18 years, singleton, no major fetal anomalies	Mean age 31 years 100% Hispanic for group 7% CHTN	BMI: not stated GWG: not stated GWG goals: not stated	Adapted CP for GDM with 4 sessions after GDM diagnosis
Kominiarek 2017 [33]	Retrospective cohort 2009–2015	US Greenville Health System, South Carolina	Exclusion: high risk medical conditions, multiple gestations, pre-pregnancy BMI > 45 kg/m^2^, entry to prenatal care after 24 weeks.	Inclusion: Pre-pregnancy height and weight and final pregnancy weight available, eligible for Medicaid coverage at the time of delivery Exclusion: Fetal demise, delivery outside of Greenville Health System	Mean age 23–26 years 22–24% Hispanic Mean pre-pregnancy BMI 26 kg/m^2^ 16–18% tobacco use	BMI: self-reported pre-pregnancy weight GWG: weight at delivery minus self-reported pre-pregnancy weight GWG goals: 2009 IOM	CP

*RCT* Randomized controlled trial, *BMI* Body mass index, *PNC* Prenatal care, *CP* CenteringPregnancy™, *GWG* Gestational weight gain, *GDM* Gestational diabetes, *CHTN* Chronic hypertension, *IOM* Institute of medicine, *CNM* Certified nurse midwife, *CD* Cesarean delivery, *LBW* Low birth weight, *SLE* Systemic lupus erythematosus

### Risk of bias of included studies

The validated Downs scale for randomized and observational studies was used to assess the quality of the studies with scores ranging from 9 to 19 with a median value of 12 (interquartile range 12–16). (Table 2) Of the 15 studies selected for this systematic review, 6 were not included in the meta-analysis because they reported only a mean gestational weight gain without regard to the participants’ pre-pregnancy body mass index [22, 23, 26, 29–31]. Two of these studies also did not report the variance of the gestational weight gain measure [29, 31]. Without a pre-pregnancy weight value, there is limited clinical interpretation for a mean gestational weight gain. Four of the six studies also did not specify if Institute of Medicine guidelines were used to counsel participants on overall gestational weight gain goals [23, 24, 30, 31].

**Table 2:** Quality Score for 15 Studies According to Threats to Validity and Overall Score

Author, Year	Threats to validity ^a^					Total
	Reporting (11)	External validity (3)	Internal validity (7)	Confounding or selection bias (6)	Power (1)	
Randomized trials						
Kennedy 2011 [29]	9	2	3	5	0	19
Harden 2014 [22]	5	0	2	2	0	9
Magriples 2015 [25]	9	1	2	5	0	17
Cohort studies						
Klima 2009 [26]	6	1	3	2	0	12
Holbrook 2010 [31]	3	1	3	2	0	9
Trudnak 2013 [27]	7	1	4	2	0	14
Tanner-Smith 2014 [35]	8	1	4	3	0	16
Zielinski 2014 [34]	8	2	0	2	0	12
Walton 2015 [28]	7	2	1	2	0	12
Trotman 2015 [24]	6	2	2	2	0	12
Mazzoni 2015 [23]	8	1	3	2	0	14
Brumley 2016 [36]	7	0	3	2	0	12
O’Donnell 2016 [32]	8	1	4	3	0	16
Schellinger 2016 [30]	5	1	2	2	0	10
Kominiarek 2017 [33]	9	1	4	3	0	17

^a^Numbers in parenthesis represent maximum score for the category

### Synthesis of results

Table 3 describes the sample size, weight gain outcomes, how the control group was chosen if applicable, any adjustments for gestational age in the analysis, total number of prenatal visits, and provider types for all 15 studies. The six studies not included in the meta-analysis are summarized as follows [22, 23, 26, 29–31]. Of note, only two studies had gestational weight gain as a primary outcome [22, 29] and only one study adapted the content of care to target weight gain [22]. A retrospective cohort study based in a clinic which served predominantly low-income minority women was the first to report on gestational weight gain in CenteringPregnancy™ from 2004 to 2006. This study found increased mean gestational weight gain in women in CenteringPregnancy™ (*n* = 61) compared to women in traditional prenatal care (*n* = 207) (32.2 pounds vs. 28.5 pounds *p* < 0.05), yet the pre-pregnancy body mass index was not reported so the proportion of women who met the gestational weight gain goals is not known [26]. Similarly, Holbrook evaluated gestational weight gain among 100 Spanish-speaking women receiving group (based on CenteringPregnancy™ model) or traditional prenatal care in 2009 in the U.S via a “pilot chart review”; however, pre-pregnancy body mass and statistical comparisons for gestational weight gain were not provided [31]. A RCT in a military setting from 2005 to 2007 found no difference in mean gestational weight gain between CenteringPregnancy™, but the authors also did not specify the pregnancy body mass index [29].

**Table 3:** Gestational weight gain outcomes for 15 studies

Author, Year	Group PNC GWG^a^	Traditional PNC GWG^a^	*p*-value or OR (95% CI)	Selection of controls and analysis details	Preterm births	GA at delivery or preterm birth in GWG analysis	Total Number of PNC visits	Provider types
Kennedy 2011 [29]	*n* = 162 33 pounds (mean)	*n* = 160 33.6 pounds (mean)	*p* = 0.71	RCT	7–10%	No correction for GA at delivery	12.9% group vs. 46.9% < 9 visits, *p* < 0.001	Physicians, midwives, and NP for both
Harden 2014 [22]	*n* = 8 5.29 ± 5.33 kg	*n* = 8 8.64 ± 3.88 kg	*p* < 0.01	“randomly assigned”	Not stated	No correction for GA at delivery	Not stated	Physicians for group
Magriples 2015 [25]	*n* = 495 48.8% excessive	*n* = 489 51.6% excessive	*p* > 0.05^e^	Secondary RCT	Not stated	Multilevel modeling accounted for variability in timing of delivery	9.3 group vs. 8.9, “not significant”	Physician or midwife for groups
Klima 2009 [26]	*n* = 61 32.2 ± 13.6 pounds	*n* = 207 28.5 ± 15.6 pounds	*p* < 0.05	All women who delivered at same hospital during study period	11–13%	No correction for GA at delivery	9.7 ± 2.7 group vs. 8.3 ± 3.4, *p* < 0.05	CNM for both
Holbrook 2010 [31]	*n* = 50 24 pounds (mean)	*n* = 50 28 pounds (mean)	“Not significant”	“convenience sample of the most recent 100 prenatal panel”	Not stated	No correction for GA at delivery	Not stated	Not stated
Trudnak 2013 [27]	*n* = 247 15.5% below healthy 35.6% healthy 41.3% above healthy 2% missing	*n* = 240 33.4% below healthy 31.3% healthy 29.6% above healthy 3.8% missing	*p* < 0.01^b^ aOR = 1.45 (0.79–2.62)^c^ aOR = 0.41 (0.22–0.78)^d^	Matched for Hispanic ethnicity, primary language Spanish, month/year of prenatal care entry	2.1–5.7%	No correction for GA at delivery	91.9% group vs. 63.8% adequate APNCU index, *p* < 0.01	Not stated
Tanner-Smith 2014 [35]	*n* = 242 25.5 ± 13.99 pounds 30.2% low 33.5% healthy 36.4% excessive	*n* = 327 21.32 ± 14.50 pounds 44.0% low 29.4% healthy 26.6% excessive	Not stated^e^	Propensity score matching for age, race, Spanish language speaker, education level, marital status, government insurance, current employment, gravidity, height, GA and weight at entry to care, pre-pregnancy BMI, systolic blood pressure, histories of non-gestational DM, depression, drug use, gynecological surgery, HTN, kidney problems, operations, blood transfusions, trauma	8–16%	Accounted for GA at delivery with multiplicative interaction terms	17.03 ± 5.83 group vs. 8.38 ± 4.13, no statistics in the unmatched sample	1 CNM and 1 physician for group
Zielinski 2014 [34]	*n* = 173 33.1 pounds (mean) 22% low 25% met 53% exceeded	*n* = 170 33.7 pounds (mean) 23% low 28% met 49% exceeded	*p* = 0.84 (mean) *p* = 0.24 (category)	Propensity score matching for age, insurance, race from *n* = 1427 women	5.8–5.9%	No correction for GA at delivery	14.2 ± 7.2 group vs. 13.4 ± 10.7, *p* = 0.27	CNM for both
Walton 2015 [28]	*n* = 202 14.9 ± 6.53 kg 52.7% excessive	*N* = 202 15.9 ± 6.53 kg 61.9% excessive	*p* = 0.11 (mean) *p* = 0.07 (category)	Selected from 2011 to 2013	5.5–6.9%	No correction for GA at delivery	“9 group visits”	CNM for both
Trotman 2015 [24]	*n* = 50 2.0% met	*n* = 50 38.0% met single provider *n* = 50 38.0% met multiple provider	*p* = 0.02 (single provider) *p* = 0.02 (multiple provider)	Selected from either single or multiple provider according to age, time, and delivery criteria	10–16%	No correction for GA at delivery	62% group vs. 40.8–51.9% attended 100% of appointments	CNM or physicians for group
Mazzoni 2015 [23]	*n* = 62 19.2 ± 13.0 pounds 3rd tri weight gain 6.7 ± 7.0 pounds	*n* = 103 18.0 ± 15.0 pounds 3rd tri weight gain 7.3 ± 6.6 pounds	*p* = 0.57 (total) *p* = 0.55 (3rd tri)	Women with GDM who delivered in 2012 at same hospital	3–5%	No correction for GA at delivery	12.4 ± 2.2 group vs. 14.0 ± 4.3 scheduled appointments, *p* = 0.002	Obstetrician, CNM, psychologist, medical assistant for group; Obstetrician or MFM specialist for traditional
Brumley 2016 [36]	*n* = 65 32.8 ± 10.7 pounds 33.8% met	*n* = 130 31.4 ± 12.7 pounds 36.2% met	*p* = 0.18 (mean) *p* = 0.24 (category)	Matched for age and pre-pregnancy BMI in 1:2 ratio	1.5–6%	No correction for GA at delivery	Not stated	Midwives for group
O’Donnell 2016 abstract only [32]	*n* = 125 46.4% excess	*n* = 2873 43.3% excess	*p* = 0.49	Women who declined CP	Not stated	Not stated	Not stated	Not stated
Schellinger 2016 [30]	*n* = 203 9.3 ± 4.5 kg	*n* = 257 10.2 ± 6.7 kg *n* = 120 (Hispanic women) 10.3 ± 5.7 kg	*p* = 0.21 (all women) *p* = 0.26 (Hispanic women)	Women who declined CP	8–11%	No correction for GA at delivery	Not stated	Health educator, diabetic educator and physician for group
Kominiarek 2017 [33]	*n* = 2117 30 pounds (18–18) median (IQR) 20% low 25% met 55% excessive	*n* = 4234 28 pounds (20–40) median (IQR) 26% low 26% met 48% excessive	*p* < 0.001 *p* < 0.001 (category)	Matched 1:2 with the next 2 women in traditional PNC who delivered with the same payer type, within 2 kg/m^2^ pre-pregnancy BMI units, and within 2 weeks of gestational age at delivery	5–7%	Weekly rate of GWG calculated and then multiplied by 40	13.6 ± 3.2 group vs. 10.3 ± 3.9, p < 0.001	NP or CNM for group

*RCT* Randomized controlled trial, *BMI* Body mass index, *PNC* Prenatal care, *CP* CenteringPregnancy™, *DM* Diabetes mellitus, *GDM* Gestational diabetes mellitus, *HTN* Hypertension, *OR* Odds ratio, *GWG* Gestational weight gain, *APNCU* Index adequacy of prenatal care as described by Kotelchuck 1994 [48]. *GA* Gestational age, *NP* Nurse practitioner, *MFM* Maternal fetal medicine

^a^Gestational weight gain reported a mean ± SD, median (IQR), or n% as a categorical variable (e.g., inadequate, adequate, or excessive gestational weight gain) depending on how the variable was reported

^b^X^2^ value for overall comparison

^c^Comparison between above and healthy weight gain

^d^Comparison between below and healthy weight gain

^e^Comparisons of gestational weight gain outcomes in group vs. traditional prenatal care in unadjusted analysis either showed an increase in excessive gestational weight gain or the statistics were not stated, but findings from either multilevel modeling or propensity score matching showed a decrease in excessive gestational weight gain in group vs. traditional prenatal care

Harden et al. reported on group-based lifestyle sessions within prenatal care in two “feasibility pilot studies with random assignment” that aimed to limit excessive gestational weight gain [22]. In the first study, women with a pre-pregnancy body mass index ≥30 kg/m^2^ were randomly assigned to either standard of care (*n* = 8) or the intervention (n = 8). Women in the control group gained significantly more weight at the 20th week of the program compared to those in the intervention (8.6 ± 3.9 kg vs. 5.3 ± 5.3 kg, *p* < 0.01). The investigators received positive feedback from both providers and patients about the program, but because the participants suggested that the medical appointments be conducted separately from the group-based lifestyle sessions, they conducted a follow-up study whereby women of all body mass index categories were randomized to either the group-based lifestyle sessions (*n* = 28) or the control group (*n* = 23) which consisted of individual goal setting, didactic nutritional education, and a 30 min standard exercise class. The group-based lifestyle sessions were similar to the initial study and also included discussion of barriers and potential solutions to reaching goals, sharing of baked snacks from the provided recipe book, exchanging of telephone numbers, and group exercises such as a walking track, dance, and prenatal yoga. Because the follow-up study occurred separately from prenatal care visits, the information was not included in the meta-analysis. Furthermore, 19 (37%) of all participants were missing gestational weight gain, so the finding that 36% in the group-based lifestyle sessions vs. 13% in the control group met the gestational weight gain goals (*p* = 0.06) needs to be interpreted with caution [22].

The two other studies not included in the meta-analysis specifically targeted women with gestational diabetes. Mazzoni et al. aimed to compare the progression of gestational diabetes (from diet to medically-treated) in group and traditional prenatal care in a prospective observational study in the U.S [23]. Participants started the program between 26 and 32 weeks gestation for a total of four sessions which covered topics such as meal planning, glucose log review, mindful eating, and lifelong diabetes prevention. Mean gestational weight gain was similar in both group (19.2 ± 13.0 pounds) and traditional (18.0 ± 15.0 pounds) prenatal care, *p* = 0.57. Lastly, Schellinger et al. developed an adapted CenteringPregnancy™ model in Spanish only for Hispanic women with gestational diabetes. The CenteringPregnancy™ model was adapted to a total of four visits in the 3rd trimester with facilitated discussions on blood sugar monitoring, nutrition, and exercise [30]. Gestational weight gain, a secondary outcome in their retrospective cohort study, was compared to women in traditional prenatal care with gestational diabetes, but only mean values were reported (9.3 ± 4.5 pounds vs. 10.2 ± 6.7 pounds, *p* = 0.26) [30].

**Fig. 2:**
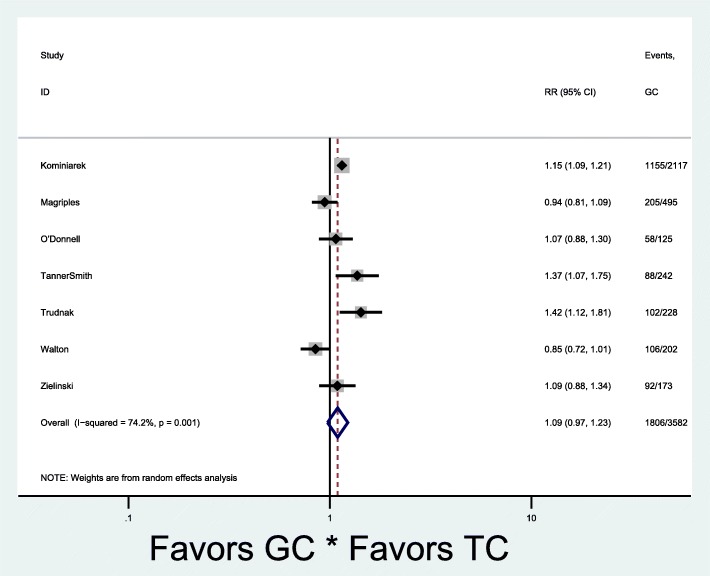
Forest plot for excessive gestational weight gain in group vs. traditional prenatal care. RR risk ratio CI confidence interval TC traditional care GC group care

**Fig. 3:**
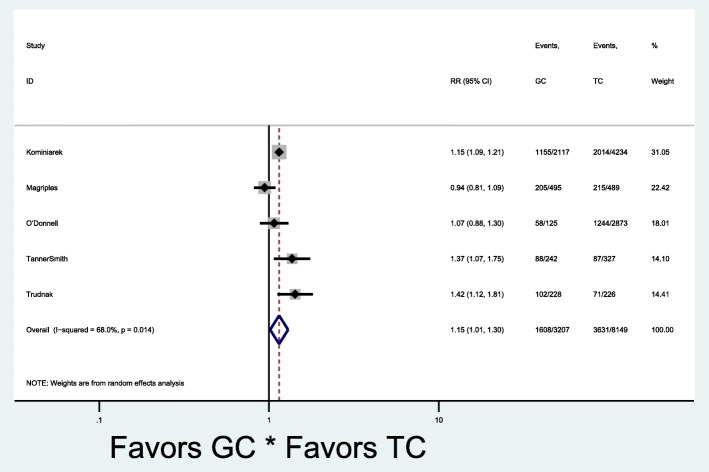
Forest plot for excessive gestational weight gain in group vs. traditional prenatal care in high quality studies. RR risk ratio CI confidence interval TC traditional care GC group care

**Fig. 4:**
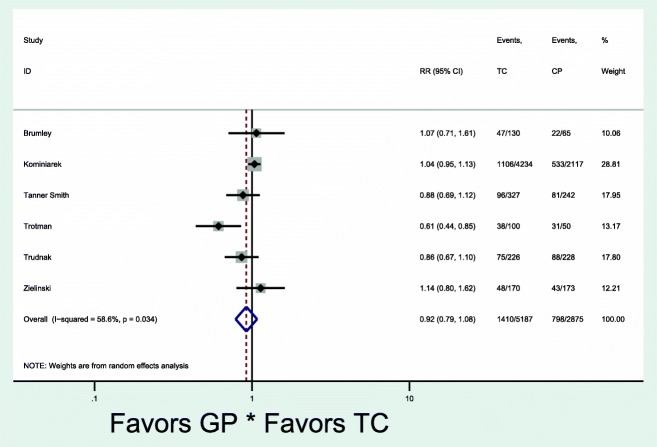
Forest plot for adequate gestational weight gain in group vs. traditional prenatal care. RR risk ratio; CI confidence interval TC traditional care GC group care. Of note, individual studies referred to gestational weight gain as “normal”, “healthy”, or “met goals”, but for the purposes of this analysis, they were grouped into the category of “adequate” gestational weight gain.

**Fig. 5:**
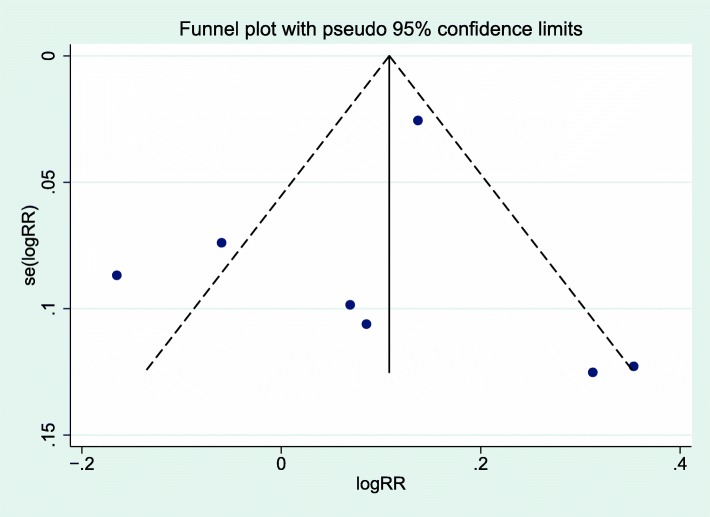
Funnel plot with 95% confidence limits for the effect of group vs. traditional prenatal care on excessive gestational weight gain. RR risk ratio

Of the nine studies in the meta-analysis, all used the CenteringPregnancy™ model and three studies had both excessive and adequate gestational weight gain outcomes. Seven studies reported the primary outcome of excessive gestational weight gain (Fig. 2) [25, 27, 28, 32–35]. Overall, there was no significant difference in the occurrence of excessive gestational weight gain [7 studies: pooled rates 47% (1806/3582) vs. 43% (3839/8521), RR 1.09, 95% CI 0.97–1.23] in group and traditional prenatal care. Given the low overall quality values (fair to poor), we separately evaluated studies in the top tertile quality scores based on the range and distribution of scores and found that excessive gestational weight gain was higher in group compared to traditional care [pooled rates 45% (1608/3207) vs. 39% (3631/8149), RR 1.15, 95% CI 1.01–1.30] (Fig. 3). There was significant heterogeneity between the studies that evaluated excessive gestational weight gain, as demonstrated by I^2^ values of 74.2% (*p* = 0.001) and 68.0% (*p* = 0.014) (Figs. 2 and 3). Six studies reported the secondary outcome of adequate gestational weight gain (Fig. 4) [24, 27, 33–36]. There also was no significant difference in adequate gestational weight gain [6 studies: pooled rates 31% (798/2875) vs. 30% (1410/5187), RR 0.92, 95% CI 0.79–1.08] in group and traditional prenatal care. There also was significant heterogeneity between the studies that evaluated adequate gestational weight gain, as demonstrated by an I^2^ value of 58.6% (*p* = 0.03) (Fig. 4). There appeared to be symmetry with the funnel plot and therefore publication bias was minimal (Fig. 5).

## Discussion

### Main findings

This systematic review found 15 studies of poor to fair quality with 13,779 subjects meeting the criteria of comparing gestational weight gain according to a group vs. traditional prenatal care model, with the number of studies further limited for the meta-analysis of excessive (*n* = 7) and adequate (*n* = 6) gestational weight gain [22–36]. We found that group prenatal care was not associated with excessive or adequate gestational weight gain except in the highest quality studies in the meta-analysis, which had a very modest but increased risk for excessive gestational weight gain for group prenatal care. Of the studies included in this meta-analysis, Tanner-Smith et al. and Magriples et al. both found reductions in excessive gestational weight gain in adjusted analysis (propensity score matching for 20 maternal characteristics and multi-level modeling, respectively) for CenteringPregnancy™ women, which suggests other confounding factors such as race-ethnicity, gravidity, pre-pregnancy body mass index, and gestational age at the entry to prenatal care are responsible for the relationship between prenatal care model and gestational weight gain [25, 35]. These two studies used the 2009 Institute of Medicine definitions for body mass index and gestational weight gain, directly compared CenteringPregnancy™ and traditional prenatal care, and had a greater total number of subjects (*n* = 569 and 984, respectively) than most studies. Conversely, Kominiarek et al. found a direct association between excessive gestational weight gain and group prenatal care, primarily among normal and overweight women, in a study of 6351 women [33].

### Strengths and limitations

Our study has several strengths. First, we used a predesigned protocol with a comprehensive search strategy conducted by a trained, expert research librarian, and two authors independently extracted data, which reduced bias. Second, we used a pre-specified sensitivity analyses according to the risk of bias assessment. Third, we pooled data from studies using a more conservative random-effects model to account for clinical heterogeneity between studies. Finally, because gestational weight gain is an important construct that impacts maternal and offspring outcomes and there are many variations of group prenatal care across the U.S., this systematic review and meta-analysis sheds some light on the state of the inconsistent evidence for group prenatal care as it relates to this important outcome. We acknowledge several limitations to this systematic review and meta-analysis. The majority of the studies were secondary analyses from RCT or retrospective cohort studies where the risk for selection bias and confounding is greater especially since women typically opt-in or self-select for group prenatal care. For example, women in group prenatal care are typically younger, of lower parity, and more frequently minorities of lower socioeconomic status, all of which can positively or negatively influence gestational weight gain [37]. The selection of women from traditional prenatal care varied among the studies and included matching for age, delivery date, ethnicity, and/or pre-pregnancy body mass index, matching for women who declined group prenatal care, and matching according to a propensity score. We noted a greater number of nulliparas [24, 27, 28, 36] and minority women [24] in group care compared to traditional prenatal care in some of the studies included in this systematic review. In summary, these factors, including participant demographics (age, race-ethnicity, geographical location), participant characteristics (age, body mass index, gestational diabetes), and intervention dose (length and number of visits, gestational age at entry) may explain the heterogeneity we observed in the meta-analysis.

We were not able to evaluate the content (i.e., how much emphasis placed on gestational weight gain goals) or dose (i.e., number of group sessions attended) of the group prenatal care program or the experience or effectiveness of the group prenatal care facilitator. The primary outcome, gestational weight gain, was not well defined in several studies and also varied from a difference between a first and last prenatal visit to a difference between the start and end of the intervention. Other limitations of these individual studies for a meta-analysis include lack of pre-pregnancy body mass index information, differing statistical approaches, and small cohort sizes.

### Comparison with existing literature

With respect to weight management, highly effective components of weight management interventions include calorie and physical activity goals, meal replacements, daily self-weighing and monitoring of food intake, behavior therapy, and frequent provider-patient contact [13]. Several of these components are already integral elements of group prenatal care such as CenteringPregnancy™. In general, group health care visits have the potential to improve the effectiveness and efficiency of health care because multiple patients are seen in the same clinical setting by a multidisciplinary team consisting of physicians, nurses, dieticians, and other health educators [38, 39]. Weight management is just one example where group care models are practiced among others including diabetes, smoking cessation, geriatrics, and osteoarthritis [40–42].

Social support in the context of group meetings with either peers or self-identified family and friends has been studied in weight management interventions that have highly effective components. A systematic review of five RCT with a group intervention found improved weight change within 1 year for the participants in the group compared to usual care [weighted mean difference − 1.4 kg (− 2.7/− 0.1 kg)] with a greater effect seen when a financial incentive was incorporated [weighted mean difference − 2.8 kg (− 5.4/− 0.2 kg)] and when the groups were led by psychologists as compared to nutritionists [9]. Furthermore, an adapted version of the Diabetes Prevention Program showed that participants in either large (≥16 members) or small groups (< 16 members), still had weight loss outcomes (5.1–5.8 kg) similar to the goals of the initial study (7% weight loss from initial weight) [43]. Commercial weight loss programs promote the concept of social support as critical to achieving goals [44, 45]. It has been proposed that support from attending meetings [e.g., Weight Watchers® and TOPS Club, Inc.® (Taking Off Pounds Sensibly)] enhances feelings of control and confidence and consequently group-based interventions result in greater weight loss compared to individual care [9, 10, 44–46]. For example, in a prospective, 2-year clinical trial that randomly assigned participants to either Weight Watchers® meetings or the self-help method, those assigned to Weight Watchers® meetings lost and kept off significantly more weight [44]. However, findings regarding social support and weight loss are mixed as another meta-analysis of 21 studies concluded that couples programs were effective in short-term weight loss, but not in long-term weight loss maintenance [47]. Given that social support is cited as a successful element in weight management programs in non-pregnant populations and the social support element that accompanies many group prenatal care models such as CenteringPregnancy™, it is reasonable to evaluate group prenatal care models regarding their effectiveness in changing health behaviors and achieving gestational weight gain goals.

## Conclusions and implications

Interventions to prevent excessive gestational weight gain are potentially most effective when they parallel effective behavioral lifestyle programs in non-pregnant populations [13]. As such, further research is needed to determine: (1) how social support influences gestational weight gain in health behavior interventions especially in groups that may vary by race-ethnicity and other maternal characteristics, (2) if adaptations to group care are needed to emphasize gestational weight gain goals and its accompanying adverse perinatal and long-term maternal outcomes such as diabetes and cardiovascular disease especially since we found increases in gestational weight gain in group prenatal care for the highest quality studies, and (3) how best to incorporate the prenatal care into the health behavior intervention so that providers can effectively communicate health risks and goals. These are important concepts because so few studies have been able to demonstrate that health behavior interventions improve gestational weight gain and other health outcomes. In conclusion, gestational weight gain for women in group prenatal care has inconsistent findings, but overall we found no differences in gestational weight gain outcomes in group compared to traditional prenatal care. We propose that prenatal care models (e.g., group vs. traditional) should be evaluated in a more rigorous fashion by including more RCT that clearly define and primarily evaluate gestational weight gain outcomes.

## Additional file

Additional file 1: Search strategy for article selection for the systematic review. This file contains the information that the librarian (S.A.F.) used to complete the article searches for the systematic review. (DOCX 14 kb)

## Abbreviations

CI: Confidence intervals
RCT: Randomized controlled trials
RR: Relative risks
TOPS: Taking Off Pounds Sensibly
