# Supplementary material for: Gestational weight gain and group prenatal care: a systematic review and meta-analysis
Source: BMC Pregnancy Childbirth. 2019 Jan 9;19:18. doi: 10.1186/s12884-018-2148-8 (PMC6327616; doi:10.1186/s12884-018-2148-8)
Supplement: Supplementary file 1 — Search strategy for article selection for the systematic review. This file contains the information that the librarian (S.A.F.) used to complete the article searches for the systematic review. (DOCX 14 kb) [file 12884_2018_2148_MOESM1_ESM.docx]

**Prepared by:**

Susan A. Fowler, MLIS

Bernard Becker Medical Library

Washington University in St. Louis

**Methods section text:**

The published literature was searched using strategies created by a medical librarian for the concepts of centering pregnancy and maternal weight gain. These strategies were established using a combination of standardized terms and key words, and were implemented in Medline 1946-, Embase 1947-, Scopus 1823-, Cochrane Database of Systematic Reviews, Database of Abstracts of Reviews of Effects, Cochrane Central Register of Controlled Trials, and Google Scholar. We also searched ClinicalTrials.gov and located 4 registered studies. All searches were completed in October and November 2016. No database supplied limits were applied. All literature database and Google Scholar results were exported to EndNote. We used the automatic duplicate finder in EndNote and 126 duplicates were assumed to be accurately identified and remove. An additional 33 duplicate citations were identified by hand and removed for a total of 377 unique citations. Searches were updated in April 2017 for an additional 81 unique citations and 2 new studies registered in ClinicalTrials.gov. Fully reproducible search strategies are provided.

Ovid Medline
Date Searched: 11/03/2016
Number of Results: 69

Reran: 4/17/2017
Applied Database Supplied Limits: 2016 – Current
Number of Results: 3

Full Search Strategy:

(centeringpregnancy.mp. OR (Centering ADJ2 Pregnancy).mp. OR (Group ADJ2 Prenatal Care).mp. OR ((group processes/ or peer group/) AND (Prenatal Care/)) OR (((Group*) ADJ2 (Process* OR Meeting* OR Think* OR care OR discussion* OR Support* OR dynamic* OR interaction*)).mp. AND ((Prenatal OR ante natal OR antenatal) ADJ2 (Care OR control))).mp.) AND (Weight Gain/ OR ((maternal or women or woman or mother* or gestational or pregnan*) adj6 (weigh*)).mp.)

Embase
Date Searched: 11/03/2016

Number of Results: 117

Reran: 4/17/2017
Applied Database Supplied Limits: Records Added 1/11/2016 – 4/17/2017
Number of Results: 10

Full Search Strategy:

(centeringpregnancy OR centering NEAR/2 pregnancy OR group NEAR/2 'prenatal care' OR ('group dynamics'/exp OR 'group dynamics' OR 'group therapy'/exp OR 'group therapy' AND ('prenatal care'/exp OR 'prenatal care')) OR group* NEAR/2 (process* OR meeting* OR think* OR care OR discussion* OR support* OR dynamic* OR interaction*) AND (prenatal OR 'ante natal' OR antenatal) NEAR/2 (care OR control)) AND ('weight gain'/exp OR (maternal OR women OR woman OR mother* OR gestational OR pregnan*) NEAR/6 weigh*)

Cochrane
Date Searched: 11/03/2016

Number of Results from each database in Cochrane

CDSR: 53
CENTRAL: 38

DARE: 1

Reran Search:4/17/2017

Applied Database Supplied Limits: 2016 - Current

Number of Results from each database in Cochrane

CDSR: 11
CENTRAL: 9

DARE: 0

Full Search Strategy:

(centeringpregnancy OR (Centering NEAR/2 Pregnancy) OR (Group NEAR/2 “Prenatal Care”) OR (([mh ^“group processes”] or [mh ^“peer group”]) AND ([mh “Prenatal Care”])) OR (((Group*) NEAR/2 (Process* OR Meeting* OR Think* OR care OR discussion* OR Support* OR dynamic* OR interaction*)) AND ((Prenatal OR “ante natal” OR antenatal) NEAR/2 (Care OR control)))) AND ([mh “Weight Gain”] OR ((maternal or women or woman or mother* or gestational or pregnan*) NEAR/6 (weigh*)))

Scopus
Date Searched: 11/04/2016

Number of Results: 96

Reran Search: 4/17/2017

Applied Database Supplied Limits: 2016-2017

Number of Results: 11

Full Search Strategy:

(TITLE-ABS-KEY((centeringpregnancy) OR (Centering W/2 Pregnancy) OR (Group W/2 “Prenatal Care”)) OR TITLE-ABS-KEY(((Group*) W/2 (Process* OR Meeting* OR Think* OR care OR discussion* OR Support* OR dynamic* OR interaction*)) AND ((Prenatal OR “ante natal” OR antenatal) W/2 (Care OR control)))) AND TITLE-ABS-KEY((maternal or women or woman or mother* or gestational or pregnan*) W/6 (weigh*))

ClinicalTrials.gov
Date Searched: 11/04/2016

Number of Results: 4
Reran: 4/17/2017 (date limited to 2016 – present)
Number of results: 2

Search Terms: (centeringpregnancy OR "centering pregnancy" OR "group prenatal care") AND “weight gain”

Google Scholar
10/27/2016 - 177 results
4/17/2017 (date limited to 2016-2017): 51

("gestational weight gain" OR "maternal weight gain") AND ("group prenatal care" OR "centering pregnancy" OR "centeringpregnancy") -"MultiCare Health System Tacoma WA Keywords"
